# Is weak CD4+ gain in the course of suppressive combination antiretroviral therapy for HIV infection a current clinical challenge? A case report and brief review of the literature

**DOI:** 10.1186/s12879-017-2942-3

**Published:** 2018-01-05

**Authors:** Camilla Tincati, Esther Merlini, Antonella d’Arminio Monforte, Giulia Marchetti

**Affiliations:** Department of Health Sciences, Clinic of Infectious Diseases and Tropical Medicine, ASST Santi Paolo e Carlo, University of Milan, San Paolo Hospital, Via di Rudinì 8, 20142 Milan, Italy

**Keywords:** CD4+ recovery, T-cell activation, IL-7, Microbial translocation, Microbiota

## Abstract

**Background:**

Individuals lacking immune recovery during suppressive cART will still represent a clinical issue in the years to come, given the high proportion of HIV-infected subjects introducing therapy late in the course of disease. Understanding the mechanisms underlying poor CD4+ T-cell gain is crucial for the correct clinical management of individuals in this context.

**Case presentation:**

An HIV-infected subject with poor CD4+ T-cell gain in the course of suppressive antiretroviral therapy was extensively investigated to identify the mechanisms behind inadequate CD4+ reconstitution. In particular, we studied the phenotype of circulating T-cells, interleukin-7 signaling in peripheral blood and bone marrow, gut function and microbial translocation markers as well as the composition of the faecal microbiota. Numerous therapeutic interventions ranging from antiretroviral therapy intensification to immunotherapy and anti-hepatitis C virus treatment were also employed in order to target the possible causes of poor immune-recovery.

**Conclusions:**

Poor CD4+ T-cell gain on suppressive antiretroviral therapy is multifactorial and thus represents a clinical challenge. Clinicians should investigate subjects’ immune profile as well as possible causes of chronic antigenic stimulation for the administration of the most appropriate therapeutic strategies in this setting.

## Background

A low CD4+ T-cell nadir upon combination Antiretroviral Therapy (cART) introduction in Human Immunedeficiency Virus (HIV) infection is linked to weak CD4+ T-cell gain [1], exposing subjects to the increased risk of clinical events [2]. Current guidelines recommend initiation of treatment early in the course of disease [3, 4], yet data from numerous study cohorts point to the steadiness of late diagnosis of HIV and thus cART start in up to 50% of infected subjects [5]. In line with these observations, it is conceivable that individuals lacking immune recovery during suppressive cART will still represent a clinical issue in the years to come. The understanding of the mechanisms underlying poor CD4+ T-cell increases in thus mandatory for the correct management of patients in this setting.

We report the case of a male subject with persistent lack of CD4+ T-cell recovery despite long-term cART. The patient underwent a plethora of targeted therapeutic interventions following thorough investigation of the possible underlying causes of poor immune response.

## Case presentation

The patient was diagnosed with HIV infection at the age of 31 in 1994. The main risk factor for HIV infection was previous intravenous drug use. Nadir CD4+ T-cell count was 26/μL. Hepatitis C Virus (HCV) co-infection was present (genotype 1a) and cytomegalovirus (CMV) serology was positive. No opportunistic infections were diagnosed at the time of HIV testing.

**Fig. 1:**
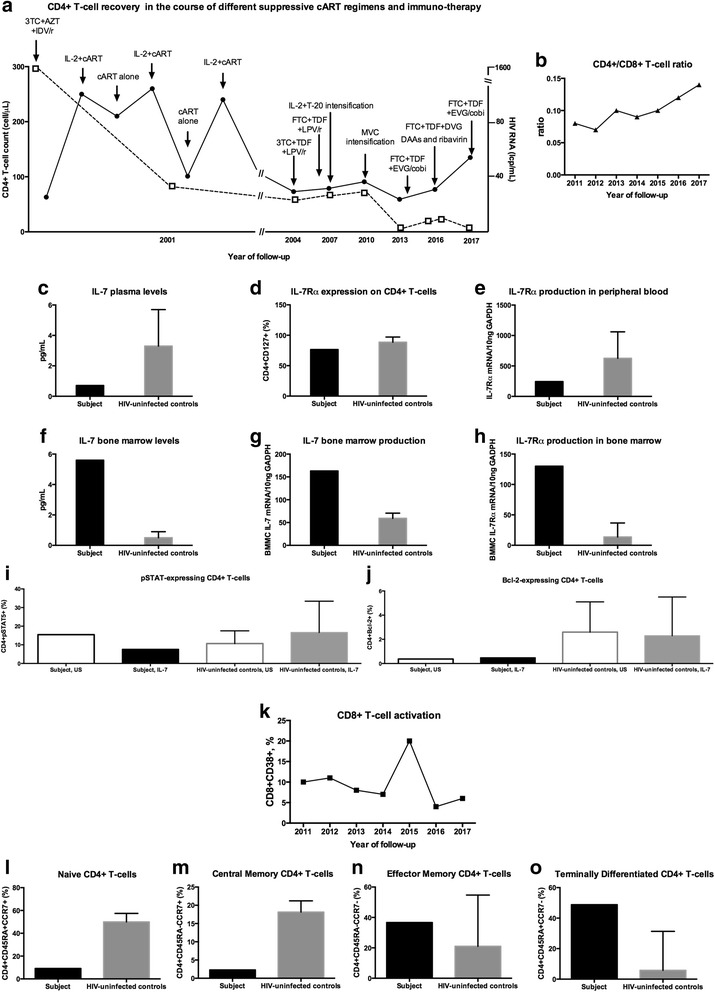
CD4+ T-cell kinetics and study of the mechanisms underlying poor immune recovery on cART. Persistent low CD4+ T-cell counts (**a**) and CD4+/CD8+ T-cell ratio (**b**) were registered in the study subject despite the administration of different suppressive cART regimens and immuno-therapy. Compared to a historical cohort of uninfected controls, the patient also displayed lower peripheral blood IL-7 levels (**c**), decreased IL-7Rα (CD127) expression on CD4 + T-cells (**d**) and IL-7Rα production in Peripheral Blood Mononuclear Cells (PBMCs) (**e**). In the bone marrow, we observed elevated IL-7 levels (**f**), increased production of IL-7 (**g**) and IL-7Rα (**h**). Lower pSTAT5- (**i**) and Bcl-2-expressing CD4+ T-cells (**j**) upon IL-7 stimulation were detected in our subject. Stable CD8+ T-cell activation (**k**) and impairment of CD4+ T-cell maturation (**l-o**) were also observed. —— represents the kinetics of CD4+ T-cell counts; −---- represents the kinetics of HIV RNA load (limit of detection: 40 cp/mL). cART: combination antiretrovrial therapy; 3TC: lamivudine; AZT: zidovudine; IDV/r: indinavir/ritonavir; IL-2: interleukin-2; TDF: tenofovir; LPV/r: lopinavir/ritonavir; T-20: enfuvirtide; FTC: emtricitabine; MVC: maraviroc; EVG/cobi: elvitegravir/cobicistat; DVG: dolutegravir; DAAs: direct acting antiretrovirals. IL-7: interleukin-7; IL-7R α: IL-7 receptor α. BBMCs: Bone Marrow Mononuclear Cells. US, unstimulated

cART was initiated with zidovudine, lamivudine and indinavir/ritonavir which was changed to tenofovir, lamivudine and lopinavir/ritonavir in 2004. Despite rapid virological suppression, poor immune recovery was observed with CD4+ T-cell counts constantly below the 200/μL threshold (Fig. 1a) and impaired CD4+/CD8+ T-cell ratio (i.e. lower than 1 [6]; Fig. 1b).

A low CD4+ T-cell count at the start of cART has been associated with clinical progression [2, 7] and hampered immune-reconstitution (reviewed in [8]; [9, 10]); of note, a low CD4+ T-cell nadir has also been shown to mirror the complex alterations of peripheral T-cell homeostasis in HIV infection, ranging from the impairment of lymphocyte maturation and function to increases in T-cell activation, death and T-regulatory cell (Treg) activity, which may not be reverted by treatment [9–20]. In accordance with these findings also a low CD4/CD8 T-cell ratio has been associated with skewed immune functions [21, 22] and increased clinical risk [6].

In 2001 the patient agreed to undergo immune-adjuvant interleukin-2 (IL-2) therapy, which at the time was being explored as a possible alternative therapeutic strategy to cART alone, given its ability to induce CD4+ T-cell increases and restore CD4+ T-specific responses to both recall and HIV antigens [23]. Three cycles of IL-2 were administered subcutaneously (1 cycle: 3 × 10^6^ IU qd, days 1–5 and 8–12) for an overall duration of 3 months. Immune-therapy accounted for transitory increases in CD4+ T-cell counts that were not, however, sustained after the interruption of IL-2 cycles (Fig. 1a, b). Furthermore, the subject was diagnosed with cutaneous VZV reactivation shortly after immune-therapy. In this respect, despite the undeniable immunological effects of adjuvant IL-2 in subjects with poor CD4+ recovery on cART [24], this strategy was abandoned for the treatment of HIV infection, given it failed to provide clinical benefit compared to antiretrovirals alone [25]. Rather, it accounted for a higher relative risk of progression to AIDS in subjects with greatest CD4+ expansion [25], finding that was linked to a sustained increase in Treg cells [26], pointing to a major pathogenic role of this subset in the clinical outcome of treated HIV disease.

Inefficient CD4+ T-cell gain during treatment has been linked to failure in de novo CD4+ T-cell production. In this respect, impaired thymic [27, 28] and bone marrow function [29–31] alongside fibrosis of secondary lymphoid organs [32, 33], may represent possible pathogenic mechanisms underlying poor CD4+ output. A bone marrow aspirate highlighting hypo-cellularity, matrix fibrosis as well as severe dysplasia of myeloid, erythroid and platelet precursors was performed in 2006, while inconclusive results were obtained from the study of thymic function through the measurement of T-Cell Receptor Excision Circles (TREC; not shown). Given the role played by IL-7 in thymopoiesis as well as in the proliferation and survival of peripheral cells through its interaction with the IL-7 Receptor (IL-7R) on thymocytes, T-cells and bone marrow macrophages [34], we decided to investigate the IL-7/IL-7R system in the study patient.

Compared to uninfected controls described elsewhere [30], the subject displayed lower IL-7 plasma levels (Fig. 1c), decreased circulating IL-7Rα (CD127)-expressing CD4+ (Fig. 1d) and IL-7Rα production in Peripheral Blood Mononuclear Cells (PBMCs) (Fig. 1e). In sharp contrast with what observed in the periphery, in the patient’s bone marrow we found increased levels and production of IL-7 (Fig. 1f, g) as well as heightened IL-7Rα expression in Bone Marrow Mononuclear Cells (Fig. 1h). Further, lower pSTAT5- (Fig. 1i) and Bcl-2-positive CD4+ T-cells (Fig. 1j) were detected following in vitro IL-7 stimulation of the patient’s PBMCs. While promising results have been produced following the use of adjuvant therapy with IL-7 in subjects with poor CD4+ T-cell recovery [35, 36], our findings warrant careful investigation of the possible reasons behind IL-7 administration failure in this setting [36], given that dysfunctional IL-7R signalling may feature discordant subjects [30, 37].

Excessive peripheral CD4+ T-cell destruction may represent another cause of hampered immune response on cART and may be due to several features strictly linked to each other: T-cell activation, ongoing viral replication/HIV persistence and chronic antigenic stimulation. In particular, aberrant T-cell activation leading to increased cell death has been constantly associated with discordant immune responses to therapy [16–18, 28, 38] and its multifactorial pathogenesis appears to be related to productive/latent HIV infection, the presence of co-pathogens (HCV, CMV) and microbial translocation.

Given that stable levels of CD8+ T-cell activation were observed over the years (Fig. 1k), in 2011 we decided to expand our knowledge on the subjects’ T-cell homeostasis by performing a thorough investigation of the CD4+ lymphocyte maturation phenotype. For this purpose, HIV-uninfected individuals were consecutively enrolled as a control group (*n* = 16; median age 31 years, IQR 28–35; female sex 69%; HCV co-infection 0%) for laboratory experiments. Our analysis revealed lower frequencies of naïve (Fig. 1l) and central memory cells (Fig. 1m) as well as higher CD4+ effector memory (Fig. 1n) and terminally differentiated lymphocytes (Fig. 1o) compared to controls, thus pointing to persistent skewing of T-cell homeostasis despite long-term virological suppression, immunotherapy and cART intensification (see below).

Data on the role of ongoing viral replication and increased reservoirs as a cause of immune activation and poor immune recovery in course of suppressive cART have been inconclusive, with proof of a relationship between such features in some studies [28, 39–41] and not in others [15, 39, 42–44]. Despite full virological suppression and no history of viral blips (Fig. 1a), in order to counteract the possible effects of persistent HIV replication below the limit of detection, in 2007 our patient underwent cART intensification with enfuvirtide in combination with additional 3 cycles of IL-2 adjuvant therapy, which did not lead to prolonged increases in CD4+ T-cell numbers (Fig. 1a). cART intensification was also carried out 4 years later with maraviroc which did not account for changes in CD4+ recovery (Fig. 1a, b), similarly to what observed in various studies evaluating the role of the CCR5-coreceptor inhibitor in subjects with discordant responses to cART [45–48], and may be due to the marginal impact of this molecule on T-cell activation in this setting [45–48]. Overall, intensification strategies with different classes of antiretrovirals have lead to modest T-cell gains [45–52] and produced controversial results regarding T-cell homeostasis [45, 46, 48, 50, 51, 53–57] and measures of HIV low level-replication/persistence [49, 51–56, 58–61]. Of note, in more recent years (2013-ongoing) no replication below the limit of detection (40 cp/mL) was measured in our subject, with the exception of two consecutive values, 11 cp/mL and 14 cp/mL, in 2016.

HIV/CMV co-infection has been described as an additional cause of discordant immune responses to cART [62], disturbing T-cell homeostasis [63, 64]. As mentioned above, the study subject displayed serologic positivity for previous CMV infection. Although the precise role of CMV reactivation in this setting is a matter of controversy [64, 65], valganciclovir administration was shown to suppress CMV DNA levels and lower T-cell activation levels [65]. Also HCV co-infection has been widely described as a factor contributing to impaired CD4+ T-cell recovery [66] and immune skewing in HIV disease [67, 68]. Paucity of data exists on the role of HCV clearance in influencing the course of CD4+ T-cell counts in subjects on long-term cART [69], however, treatment of HCV infection may have a beneficial effect on other determinants of discordant immune responses, i.e. T-cell activation [70] and liver fibrosis [69].

The patient showed mild progression in terms of HCV-related liver disease over the years and agreed to start anti-HCV therapy with dasabuvir, ombitasvir/paritaprevir/ritonavir and ribavirin at the end of 2016 (Fib-4: 1.62; liver stiffness measured by transient elastography: 7.1 kPa; fibrosis stage F2).). The subject displayed rapid HCV RNA abatement (from 22258cp/mL to undetectable levels at week 8) as well as a sustained virological response at week 24 (February 2017). CD4+ T-cell numbers showed a slight rise compared to previous years, yet the subject still displays persistent CD4 depletion (latest CD4 T-cell count and CD4/CD8, respectively, 135/μL and 0.17; Fig. 1a, b). Follow-up is currently ongoing and aside from information on the kinetics of CD4+ T-cell counts, it will be interesting to observe the long-term outcome of direct-acting antiviral agents (DAAs) on peripheral T-cell homeostasis and other markers of immune function in the absence of the modulatory effects of pegylated-interferon-α [71, 72].

Finally, microbial translocation has been extensively called upon as a cause for T-cell activation and inadequate CD4+ on cART [73–75], most likely linked to the enduring structural and anatomical defects of the gastrointestinal mucosa [76] as well as skewing of the gut microbiota, thus accounting for impaired local and systemic immunity [76] and hampered CD4+ reconstitution [77, 78]. Our subject displayed higher microbial translocation markers compared to HIV-uninfected controls (lipopolysaccharide: 459 pg/mL vs 75 pg/mL, IQR 75–79; soluble CD14: 2.2 μg/L vs 1.9 μg/L IQR 1.4–2.4), increased gut permeability parameters (lactulose/mannitole ratio: 0.03) and an outgrowth of faecal *Bacteroides intestinalis*/*Bacteroides uniformis*; further, exposure of the patient’s PBMCs to various Toll-Like Receptor bacterial agonists resulted in a down-regulation of HLA-DR/CD38 co-expression on CD8+ T-cells. These findings suggest T-cell hypo-responsiveness to subclinical endotoxemia in subjects with inadequate CD4+ recovery [73, 79] and may explain why treatment approaches targeting microbial translocation have failed to significantly reduce CD8+ T-cell activation in this setting [80].

## Discussion and conclusions

The present case report highlights the multifactorial origin of poor CD4+ T-cell gain on suppressive antiretroviral therapy thus emphasizing the difficulties of its clinical management. Given that immune failure on effective treatment may still represent a common condition in the future given delayed cART introduction [5] despite current recommendations [3, 4], we call for further research on subjects’ immune profile [20] and possible causes of chronic antigenic stimulation for the administration of appropriate therapeutic strategies in this setting.

## Abbreviations

cART: Combination antiretroviral therapy
CMV: Cytomegalovirus
DAAs: Direct-acting antiviral agents
HCV: Hepatitis C virus
HIV: Human immunedeficiency virus
IL: Interleukin
IL-7R: IL-7 Receptor
PBMCs: Peripheral blood mononuclear cells
TREC: T-Cell receptor excision circles
Treg: T-regulatory cell
VZV: Varicella Zoster Virus
